# Does baseline language ability predict response to early intervention for toddlers with early signs of autism? Evidence from a caregiver-mediated program

**DOI:** 10.3389/fnhum.2026.1704374

**Published:** 2026-03-18

**Authors:** Jessica Ann Brian, Ian Roth, Lonnie Zwaigenbaum, Isabel Smith, Lori-Ann Rosalind Sacrey, Kate Bernardi, Stacey MacWilliam, Sara Daoud, Erin Dowds, Sanne Jilderda, Samara Osten, Anna Michelis, Abbie Solish

**Affiliations:** 1 Autism Research Centre, Holland Bloorview Kids Rehabilitation Hospital, Toronto, ON, Canada; 2 Department of Paediatrics, University of Toronto, Toronto, ON, Canada; 3 Autism Research Centre, Glenrose Rehabilitation Hospital and University of Alberta, Edmonton, AB, Canada; 4 Autism Resarch Centre, IWK Health Centre and Dalhousie University, Halifax, NS, Canada

**Keywords:** autism, caregiver-mediated, communication, early intervention, naturalistic, parent-mediated, toddler

## Abstract

**Background:**

Caregiver-mediated interventions (CMIs) for young autistic children are supported by mounting evidence of efficacy. Attempts to identify child-level factors that predict treatment response have yielded inconsistent findings, with very few such studies focused on the toddler age range.

**Objective:**

Secondary analysis of data from a randomized control trial (NCT03215394) was conducted to explore predictive relationships between toddlers’ communication-related abilities and response to treatment in a CMI program. Participants. Sixty-seven toddler-caregiver dyads (62 mothers, 4 fathers, 1 grandmother) participated across three Canadian sites. Participating toddlers (52 boys, 15 girls) had a diagnosis of autism or early signs thereof and ranged in age from 18 to 32 months (*M* = 25.95 months).

**Methods:**

All dyads participated in a 12-week CMI (the Social ABCs). Communication-related skills were assessed at baseline using a direct standardized assessment of expressive language, a caregiver interview to capture receptive and expressive communication, and a parent-report inventory to document words used and understood. Analyses. A series of regressions with simple mediation analyses were performed to identify communication-related predictors of intervention change scores for caregiver fidelity and toddler outcomes. In all cases, toddler age was entered as a mediator to explore any influence on these relationships. Results. Toddlers’ functional communication inversely predicted their caregivers’ mastery of program strategies. Specifically, lower toddler functional receptive and expressive communication predicted larger gains in caregivers’ strategy use. Conversely, higher baseline (receptive but not expressive) communication-related skills predicted greater gains in toddlers’ responsivity. Trends emerged for prediction of toddler smiling and social orienting. The clinical measure selected for this study did not predict outcomes.

**Conclusion:**

Caregiver-reported information about toddler communication-related skills was informative for predicting the primary program outcomes. Findings may be helpful in informing clinical triage decisions and personalizing intervention approaches.

## Introduction

Early intervention for toddlers on the autism spectrum (formally, autism spectrum disorder / ASD) can lead to developmental progress in a range of domains including language, communication, social skills, and cognitive abilities (Dawson et al., 2010; Sandbank et al., 2020, 2023). To date, most empirical studies and systematic reviews have focused on group-level outcomes, with only a small number of studies exploring whether (or which) child-level factors predict treatment response, an evidence gap initially highlighted over a decade ago (Vivanti et al., 2014). Given substantial heterogeneity in autism presentation across individuals, it stands to reason that particular intervention elements or approaches might vary in terms of “fit” (Lai et al., 2020) across children with varying patterns of strengths, learning needs, and preferences, and at different developmental stages. For example, Koegel and colleagues proposed that “interventions may be more appropriate for subpopulations of individuals with ASD depending on their verbal levels at intake” (2020, p. 2958). The heterogeneity of presentations across the autism spectrum, together with the pressing need to provide evidence-based supports as soon as first signs emerge, establishes the importance of examining factors that predict response to intervention in the toddler years. Moreover, understanding child-level factors that predict which children will benefit most from which approaches may be informative for clinical triage processes, program planning, and advocacy (see Lord et al., 2022).

Amongst baseline child-level characteristics, cognition (IQ), adaptive functioning, and autism characteristics have been demonstrated to predict response to intervention across several studies (e.g., Frazier et al., 2021; Grzadzinski et al., 2023; Koegel et al., 2020; Landa, 2018; Perry et al., 2011; Smith et al., 2015; Vivanti et al., 2014). Pre-intervention language skills or related communication abilities also hold promise as predictors, but findings to date have been mixed. One challenge with interpretation across studies is the use of different measures that capture diverse aspects of communication. Communication entails a broad array of skills involved in sharing information with another person, including through expressive language (use of words, signs, and gestures to express oneself), receptive language (understanding another person’s message), and other modes of sharing information such as facial expressions, gaze, or tone of voice. Following Koegel et al. (2020) the term “communication-related skills” will be used in the current paper to refer broadly to language and other forms of communication. However, when discussing particular research findings or measures, the relevant specific terminology will be used wherever this level of detail is available.

Varied associations have been reported between communication-related skills and treatment response in autism intervention research. Namely, some studies report associations in a positive direction, wherein individuals who begin with stronger skills make larger gains (e.g., Casenhiser et al., 2011 – total language; Tager-Flusberg and Kasari, 2013 – spoken words; Virues-Ortega et al., 2013 – expressive language), whereas others report the opposite (e.g., Siller et al., 2013 found a larger intervention response for children with *lower* expressive language skills at baseline). Other studies have failed to demonstrate such an association (Green et al., 2010; Hardan et al., 2015) or have identified complex interactions between variables. For example, Fossum et al. (2018) identified a profile predictive of positive response to intervention that included preschoolers with high expressive language ability, positive affect, and appropriate toy contact, combined with reduced social avoidance and repetitive vocalizations. Thus, although baseline communication-related skills have potential as informative predictors, consistent patterns have yet to be firmly established. Moreover, prediction of outcomes in the younger years remains under-studied, resulting in an evidence gap with respect to interventions for toddlers.

Naturalistic developmental behavioral intervention (i.e., NDBI; Schreibman et al., 2015) approaches, which have gained traction over the past decade, provide some insights into the role of communication-related skills in early intervention with toddlers. A solid body of evidence demonstrates group-level developmental progress across studies that use NDBI approaches (e.g., see Sandbank et al., 2020, 2023; Song et al., 2025, for evidence syntheses). In efforts to understand which children are best served by NDBI models, some researchers have examined outcomes specifically for young children who enter intervention with relatively limited expressive language abilities. Two recent studies (Gengoux et al., 2019; McDaniel et al., 2020) report on outcomes from autistic children aged 2–5 years who also presented with expressive language delays (i.e., with scores 1–3 standard deviations below the mean, depending on the child’s age, as measured on the Preschool Language Scale (PLS-5); Zimmerman et al., 2011). In both studies, children participated in a 12-week NDBI, called the ‘Pivotal Response Treatment Package’ (PRT-P) which is an in-home program delivered by therapists, with associated parent training. Group-level increases were reported for children’s word use and vocal responsiveness immediately or 12 weeks following program completion (Gengoux et al., 2019; McDaniel et al., 2020, respectively), when compared to controls receiving community treatment as usual. Although these findings demonstrate positive outcomes for young children with delayed language, the predictive power of different language profiles was not examined.

Barrett et al. (2020) similarly examined outcomes from a 6-month PRT-based NDBI program (‘Pivotal Response Intervention for Social Motivation’ or ‘PRISM’ intervention) with children aged 1.5–4.5 years. Compared to waitlist controls, children in the treatment condition demonstrated gains in social responsiveness to their caregivers and increased spoken language (mean length of utterance). Notably, the intervention was found to be particularly effective for a small subgroup that the authors defined as “minimally verbal” based on having used fewer than 30 functional spoken words during an initial play interaction – this subgroup experienced particularly large increases in responsivity and word use following the intervention, demonstrating a relative advantage for children starting with fewer spoken words. These findings mirror the earlier findings by Siller et al. (2013), wherein young children with baseline expressive language abilities below a 12-month level exhibited the largest expressive language gains in response to a caregiver “capacity building” program designed to foster coordinated play between caregiver and child (‘Focused Playtime Intervention’; FPI). Taken together, these findings suggest that NDBI approaches may be particularly well-suited to children at relatively early stages of expressive language development.

The studies discussed above report on NDBI approaches that are delivered by therapists with some involvement from family caregivers (i.e., concomitant parent training is a cornerstone of NDBI models; Smith et al., 2015). However, in some NDBI models, the intervention is delivered exclusively by a family caregiver (usually a parent) who is trained to implement the intervention strategies – such a difference might uncover unique predictors. These *caregiver-mediated intervention* (CMI) approaches often focus on toddlers under 3 years old (see Zhao et al., 2025, for a review), with some studies enrolling children as young as 1 year of age (Kasari et al., 2010; Rogers et al., 2012, 2019; Stahmer et al., 2020; Yoder et al., 2021), allowing for exploration of early developmental factors. Across studies, CMI approaches appear to be acceptable and feasible (e.g., see Pellecchia et al., 2024, for a review), and they yield group-level gains in various aspects of toddler development and skill acquisition (e.g., responsiveness to caregiver, adaptive and social communication skills; see review by Zhao et al., 2025). However, as with studies in older children, the examination of individual predictors is scant in this younger age range. Moreover, “language-level” groupings that are conceptualized in studies of older children are not likely to be applicable to toddlers. For example, using a cut-off of <30 functional spoken words to identify a “minimally verbal” subgroup may not be meaningful in the first 2 years of life because “nearly all toddlers younger than about 18 months of age are NV [non-verbal] or MV [minimally verbal] … However, one could argue that limited verbal ability during the toddler years is meaningfully different from that during later childhood due to the developmental trajectory of language” (Koegel et al., 2020, p. 2958). As such, the extent to which emerging communication-related abilities predict treatment response in autistic *toddlers* is particularly understudied and remains poorly understood. Extrapolating from older samples may not yield accurate information to guide clinical decisions about who will benefit from which interventions and when. Direct examination of predictors in the toddler years has become possible with the rise of interventions developed for autistic toddlers.

One such CMI program, the Social ABCs, was developed specifically for use with toddlers (≤ age 3 years) with emerging or confirmed autism, and is delivered exclusively by a family caregiver. The standard version of the program entails 12 weeks of individual parent-coaching (tapering over time) supported by a Parent Manual. The program entails coaching caregivers in the use of 10 specific strategies to enhance child social attention and vocal communication directed to the parent, as well as positive affect-sharing between caregiver and child. Each caregiver receives live, in-the-moment coaching while interacting with their toddler, an approach that has been identified to be particularly effective (referred to as “real-time” “triadic intervention” in Sone et al., 2021). Research findings, across pilot, randomized control trial, and community implementation studies, demonstrate that participation in the Social ABCs yields increased use of enhancing strategies by caregivers (i.e., implementation fidelity) and group-level gains in toddlers’ early social communication skills (i.e., vocal responsivity, social orienting; see Brian et al., 2016, 2017, 2022a, for further details). An abbreviated, 6-week, group-based adaptation of the program has also demonstrated positive findings from a large pilot evaluation (Brian et al., 2022b). An attempt to identify predictors did not yield any informative family- or treatment-level factors, including marital status, number of languages spoken in the home, frequency of English spoken in the home, which caregiver was coached, their ethnicity, occupation, educational attainment, or the number of coaching sessions attended (note that all had attended a minimum of 5 sessions; Brian et al., 2022b). The predictive role of children’s communication-related skills before starting the program has not been explored.

### Purpose of the current study

The primary purpose was to understand the role of toddlers’ communication-related abilities on their response to the standard, 12-week, 1:1 version of the Social ABCs. Given the importance of caregiver fidelity in CMI models (i.e., all therapeutic components are delivered by the caregiver, and previous findings show associations between fidelity and child progress; Brian et al., 2022a), we also examined caregiver fidelity as an outcome. Program enrollment in the Social ABCs places no restrictions on minimal or maximal developmental levels (i.e., there were no exclusion criteria based on toddlers’ expressive language, other communication-related skills, or cognitive abilities), resulting in the inclusion of toddlers across a wide range of levels at program entry. This enables the examination of possible associations between early communication-related skills and outcomes. In light of challenges with defining a “minimally verbal” subgroup in this young age range, the current study explored communication-related skills as continuous, rather than categorical, variables. Moreover, information from multiple sources was used to capture different aspects of communication-related skills across participants (details below).

#### Hypotheses

Despite variability across findings and limited evidence from toddlers, the following hypotheses were proposed: Toddlers’ baseline expressive language skills, expressive communication, and word use will be inversely associated with change in (1) caregiver fidelity and (2) toddler vocal responsivity. Examination of receptive communication-related predictors and secondary (non-verbal) Social ABCs outcomes (social orienting and shared smiling) was exploratory.

## Methods

The current study entailed secondary analysis of data from a randomized control trial (RCT) evaluating the Social ABCs with an attention training co-intervention (Clinicaltrials.gov identifier NCT03215394). Primary intervention outcomes paralleled previous findings (Brian et al., 2017, 2022a), revealing significant group-level gains in caregiver fidelity (i.e., strategy use), and toddler responsivity, smiling, and social orienting (Brian et al., 2025). Toddlers were assessed prior to program entry (at baseline; BL) using one domain of a standardized direct clinical assessment measure, a standardized parent interview of children’s everyday communication, and a parent questionnaire of vocabulary (measures described below). Video-coded data were used to characterize the following outcomes: Caregiver implementation fidelity (primary parent outcome), and toddler responsivity (primary toddler outcome), initiations, orienting toward caregiver, and social smiling. The *change scores* (post- vs. pre-intervention) for these target skills were used analyses.

### Attention training co-intervention

Prior to beginning the Social ABCs, participants were randomly assigned to complete 4 weeks of computer-based Attention Training (AT) or a control condition (viewing child-appropriate video segments) to explore whether attention training would impact (i.e., “prime”) participants’ response to the Social ABCs. The methods and results of the AT phase are described in Sacrey et al. (2025). The main Social ABCs outcomes and the impact of AT on Social ABCs were among the primary analyses in the RCT (reported by Brian et al., 2025) but are not the focus of the current paper.

Before conducting the main analyses of interest, preliminary analyses examined whether AT had any influence on Social ABCs change scores, using non-parametric K-independent samples, to explore appropriateness for inclusion in regression analyses. In cases where regressions used change scores that differed between AT and control conditions, condition would be entered as a covariate. The impact of AT on the variables of interest was assessed using Mann–Whitney U. There was no significant difference between the two AT conditions on either of the six Social ABCs change scores (all *p*s > 0.05). As such, AT condition was not included as a covariate in the following analyses and is not included in the results and discussion below. All participants received the Social ABCs following the AT (or control) phase.

### Participants

See Table 1 for family demographic information and Table 2 for toddlers’ baseline characteristics. Sixty-seven families (62 mothers, 4 fathers, 1 grandmother) participated in a randomized control trial (RCT) of the Social ABCs across three sites in Canada (Toronto, Ontario; Edmonton, Alberta; and Halifax, Nova Scotia). Toddlers (52 boys, 15 girls) ranged in age from 18–32 months (*M* = 25.95 months), were born at 37–42 weeks’ gestation weighing ≥ 2,500 g, and had a confirmed diagnosis (*n* = 32) or clinically significant early signs of autism. Early signs were based on caregiver-reported developmental concerns and clinical judgement informed by scores on the assessment measures described below, which were conducted prior to beginning the Social ABCs (i.e., following the AT co-intervention). Ten-minute video segments were collected at two key time points: (1) During a baseline free-play interaction between caregiver and toddler, and (2) following the final week 12 Social ABCs coaching session. No coaching took place during the video collection sessions and caregivers were instructed to play or interact with their child as they typically do. All participants received the 12-week, individual Social ABCs intervention (most in-home, with a small number completing the program virtually due to COVID-19 isolation measures). Table 1 shows caregiver ethnicity (41.8% identified as Black, Indigenous, or a Person of Color (BIPOC), 44.8% White, and 11.9% did not disclose); educational attainment (high school or less: 19.4%; college or trade school: 22.4%, undergraduate university degree: 26.9%, graduate school: 13.4%); and marital status (61.2% married, 13.4% in a common law relationship, 10.5% separated or never married – none were divorced). 39.1% of families had two children, and 44.9% of the toddlers were first-born.

**Table 1:** Family and caregiver characteristics.

Caregiver/family characteristic	N (%)
Ethnicity	BIPOC^1^: 28 (41.8) White: 30 (44.8) Not disclosed: 8 (11.9) Missing: 1 (1.5)
Educational attainment	Highschool or less: 13 (19.4) College^2^/Trade school: 15 (22.4) University undergraduate degree: 18 (26.9) Graduate degree: 9 (13.4) Missing: 12 (17.9)
Marital status	Married: 41 (61.2) Common law: 9 (13.4) Separated: 3 (4.5) Never married: 4 (6.0) Missing: 10 (14.9)
Birth order of participant	Firstborn: 31 (46.3) Second: 20 (29.8) Third or later: 8 (11.9) Missing: 8 (11.9)
Number of children in family	One: 17 (25.4) Two: 27 (40.3) Three or more: 14 (20.9) Missing: 9 (13.4)

^1^BIPOC: Black, Indigenous, Person of Colour; ^2^College: In the Canadian context, “College” typically refers to post-high school diploma or certificate programs (usually ≤ 2 years), whereas “University” typically refers to a Bachelor’s degree program (usually 3–4 years).

**Table 2:** Toddler baseline characteristics.

Baseline characteristic	*N*	Mean	*SD*	Range
Toddler chronological age (CA, months)	67	25.95	3.65	18–32
ADOS-Toddler total score	62	19.90	5.92	3–28
PROCESS^1^ total score	59	22.90	9.11	4–43
MSEL^2^ EL – Age equivalent (AE, months)	63	13.79	6.97	4–36
MSEL EL – Developmental quotient (DQ)^3^	63	52.87	23.79	15–120
MSEL VR – AE (months)	63	17.81	6.63	6–46
MSEL VR – DQ	63	69.58	25.98	23–178
CDI^4^ – Words understood	60	145.80	113.29	0–393
CDI – Words spoken	60	51.45	95.97	0–377
VABS^5^ RC – AE (months)	60	11.42	6.07	0–27
VABS EC – AE (months)	61	11.88	5.53	4–26

^1^Parent-Rated Observation of Communication, Emotion and Social Skills (previously APSI). ^2^Mullen Scales of Early Learning (EL: Expressive Language; VR: Visual Reception). ^3^DQ = AE/CA × 100. ^4^MacArthur Communicative Development Inventory – Words and Gestures. ^5^Vineland Adaptive Behavior Scale-3rd edition (VABS-3); RC: Receptive Communication; EC: Expressive Communication.

### Measures

#### Video-coded variables

Video-coded data (based on 10-min toddler-caregiver free-play segments) were used to characterize caregiver implementation fidelity as well as toddler outcomes (defined below). All video coding was conducted using well-established coding procedures developed for and used in previous studies, blinded to study time point, with interrater reliability established for 20% of videos (see Brian et al., 2016, 2017, 2022a, 2022b).

##### Implementation fidelity

As outlined by Koegel and Koegel (2006) and consistent with previous work (Brian et al., 2016, 2017, 2022b), caregiver implementation fidelity was calculated via continuous interval coding, consisting of ten, 1-min intervals. Each interval was coded as correct or incorrect / not used across 10 program techniques for which the caregiver received coaching: Child choice, child attending, clear opportunity, contingent reinforcement, natural reinforcement, and reinforcement of attempts, shared control, pace, recast, and positive emotion (see Brian et al., 2017 for definitions of these strategies). Implementation fidelity is reported as percentage (%) of intervals during which the caregiver demonstrated appropriate / correct use of techniques.

##### Toddler and dyad outcomes

For the current study, we used change scores (post - pre) for the following behaviors as the dependent variables: (1) *Toddler responsivity* to caregiver-provided communication opportunities. This included any toddler vocalization, directed to the caregiver, that occurred immediately following the caregiver’s single-word cue (“model-prompt”), calculated as: % Responsivity = [(number of responses ÷ number of caregiver language opportunities) × 100]. (2) *Toddler initiations* – coded as number (rate/min) of toddler-led vocal overtures directed to the caregiver; only considered an initiation when there was no preceding directive action from the caregiver (cue, request, question) within the prior 3 s. (3) *Social orienting* – interval-coded as present when the toddler’s head was oriented toward the caregiver’s torso or head/face at any time during a 5-s coding interval. This code did not require eye-to-eye gaze. (4) *Smiling* – coded separately for toddler and caregiver, as presence or absence of a clear smile per 5-s coding interval (based on detailed video coding instructions including visual representations of smiling codes); *Shared smiling* between caregiver and toddler entailed a count of all intervals where toddler and child were both smiling. Inter-rater reliability was moderate (Kappa = 0.50 for toddler responsivity) to substantial (Kappa = 0.70, 0.66, and 0.63 for adult smile, child smile, and orienting, respectively).

#### Clinical assessment measures

*Vineland Adaptive Behavior Scales – 3rd edition* (VABS-3; Sparrow et al., 2016). The VABS-3 is a semi-structured parent interview designed to assess adaptive functioning in everyday life. Adaptive behavior is assessed across four domains – Communication, Daily Living, Socialization, and Motor skills, outlined by typical developmental milestones anchored to specific ages. As a measure of *adaptive functioning* (vs. *capacity*), the VABS-3 captures the extent to which an individual engages in activities on a regular basis (“usually,” “sometimes,” or “never”) – the tool asks whether a child *does*, rather than *can do* each of the developmentally sequenced items. The Communication domain includes a “Receptive” subdomain that characterizes an individual’s understanding and responses to communication from others, such as looking toward caregivers when they are speaking, responding to gestures and facial expressions, understanding the meaning behind someone’s tone of voice, and understanding spoken language, signs, and other communicative input. The “Expressive” subdomain captures information about making sounds, using words/phrases/sentences (spoken or otherwise), using gestures, and using facial expressions to communicate. Age-equivalent scores from the Communication domain (Receptive and Expressive subdomains) were included in the current paper.

*MacArthur Communicative Development Inventories – Words and Gestures* (CDI, Infant Scale; Fenson et al., 2007) is a highly reliable and well-validated parent-report measure that provides an inventory of the child’s current abilities. The infant scale covers the period from 8 to 16 months but has also been used to map the developmental trajectories of older autistic children (e.g., Charman et al., 2003; Iverson, 2018). The CDI subscales capture words and phrases understood, words spoken, and gestural communication. The raw scores for total words understood (CDI-WU) and total words spoken (CDI-WS) were included in the current analyses.

*Mullen Scales of Early Learning* (MSEL; Mullen, 1995) is a standardized direct assessment of children’s developmental abilities from 0 to 68 months across domains, used widely in this population for research and clinical purposes. The measure has adequate-to-good psychometric properties (e.g., internal consistency ≥ 0.90) and good test–retest reliability (≥ 0.80 across 1–2 weeks). Due to the young age of participants and their limited tolerance for lengthy assessment, we prioritized the Expressive Language (EL) and Visual Reception domains of the MSEL. For this paper, we focused on the EL domain, with scores represented as age equivalents (AE; derived from raw scores) and developmental quotients (DQ; calculated as [AE/chronological age (CA)] × 100). We elected not to use the MSEL T-scores as many children (~40% of our sample) had T-scores ≤ 20, yielding inadequate sensitivity to explore the associations of interest (i.e., the scale does not provide specific T-scores when a child’s score is < 20). The Expressive Language domain of the MSEL focuses mostly on vocal and verbal output.

The *Autism Diagnostic Observation Schedule (ADOS-2), Toddler Module* (Lord et al., 2012) was used to characterize toddlers’ autism-related profiles in the areas of social communication (Social Affect domain; SA) and repetitive /restricted /intense behaviors and/or interests (Restricted and Repetitive Behavior domain; RRB). The Toddler module has strong internal consistency for the SA domain (Cronbach’s *α* = 0.88–0.90) but poor for the RRB domain (α = 0.50); as reported by McCrimmon and Rostad (2014). The ADOS was used to characterize the sample but not examined as a predictor.

The *Parent-Rated Observation of Communication, Emotions, and Social Skills* (PROCESS©; formerly APSI; Sacrey et al., 2018; Bryson et al., 2007) was used to capture parent-reported autism-related characteristics of toddlers at the beginning of the study. This is a 26-item forced-choice questionnaire (“yes – sometimes – no”), with higher scores representing more frequent and/or marked autism-related characteristics, and age-specific cut-offs. Sensitivity and specificity for total scores at 18 months are 0.65 and 0.72 (based on a cutoff of 9; Sacrey et al., 2018). The PROCESS© questionnaire was used to characterize the sample but not examined as a predictor.

### Analytic plan

The influence of baseline communication-related skills on Social ABCs outcomes was assessed using five putative predictors: MSEL Expressive Language age equivalent (MSEL-EL-AE), VABS-3 Receptive and Expressive Communication age equivalents (VABS-RC-AE and VABS-EC-AE), and MacArthur CDI Words Understood (CDI-WU) and Words Spoken (CDI-WS). A series of regressions with simple mediation analyses were performed using the Hayes (2013) PROCESS macro to identify communication-related predictors of intervention change scores. In all cases, toddler age was entered as a mediator to see if age influenced any of these relationships. The PROCESS macro was chosen over other analytic approaches (such as Mixed Modeling) because it allows for continuous predictor variables, as well as both mediation and covariates. Baseline age was used as a mediator rather than a covariate in order to determine how or why age may influence any particular relationship (mediation) rather than removing an effect of age (covariate). Due to multiple tests, a more conservative *p*-value of ≤ 0.01 was used for significance testing. Bootstrapping with 5,000 resamples was used to calculate the 95% confidence intervals for the indirect effect. Significant effects on the variables of interest (direct predictors and those that are mediated by age) are described in detail in each section below.

## Results

### Influence of communication-related skills on social ABCs outcomes

The results of the regression analyses are summarized in Table 3. Only the significant results, and sub-threshold trends related to program objectives, are described in detail below.

**Table 3:** Summary of regression findings.

		Social ABCs outcome variable						
Predictor	Fidelity	Responsivity	Initiations	Orienting	Child Smiling			Parent Smiling
Mullen expressive language – age equivalent (MSEL-EL-AE)								
Direct	−0.57 (0.04)	0.01 (0.11)	0.002 (0.76)	−0.001 (0.80)	0.001 (0.63)			0.002 (0.55)
Age	0.18 (0.02)	0.16 (0.03)	0.17 (0.02)	0.18 (0.02)	0.18 (0.012)			0.18 (0.012)
Control Age	−0.41 (0.08)	0.02 (0.06)	0.01 (0.52)	−0.001 (0.84)	0.003 (0.17)			0.002 (0.59)
Mediation	ns	ns	ns	ns	ns			ns
Vineland receptive communication (VABS-RC-AE)								
Direct	−0.81 (<0.01)*	0.03 (<0.01)*	0.001 (0.46)	−0.009 (0.03)	0.003 (0.16)			−0.001 (0.75)
Age	0.09 (0.31)	0.06 (0.52)	0.09 (0.31)	0.07 (0.40)	0.07 (0.40)			0.07 (0.40)
Control Age	−0.72 (0.02)	0.03 (<0.01)*	0.01 (0.36)	−0.001 (0.03)	0.004 (0.09)			−0.001 (0.76)
Mediation	**21% (<0.01)**	17% (ns)	ns	ns	ns			ns
Vineland expressive communication (VABS-EC-AE)								
Direct	−1.04 (0.003)*	0.02 (0.07)	0.006 (0.55)	−0.01 (0.06)	0.003 (0.29)			−0.003 (0.27)
Age	0.16 (0.10)	0.11 (0.26)	0.14 (0.12)	0.15 (0.11)	0.15 (0.11)			0.15 (0.11)
Control Age	−0.90 (0.001)*	0.02 (0.05)	0.001 (0.41)	−0.001 (0.07)	0.004 (0.11)			−0.003 (0.28)
Mediation	**23% (<0.01)**	ns	ns	ns	ns			ns
MacArthur CDI^1^ Words Understood (CDI-WU)								
Direct	0.03 (0.02)	0.002 (0.001)*	0.001 (0.07)	−0.002 (0.35)	0.001 (0.70)			0.001 (0.78)
Age	0.007 (0.17)	0.003 (0.52)	0.005 (0.29)	0.005 (0.32)	0.005 (0.32)			0.004 (0.32)
Control Age	−0.03 (0.12)	0.002 (0.001)*	0.001 (0.05)	0.004 (0.36)	0.001 (0.43)			0.001 (0.78)
Mediation	13% (ns)	**24% (<0.01)**	ns	ns	13% (ns)			ns
MacArthur CDI Words Spoken (CDI-WS)								
Direct	0.02 (<0.01)*	0.001 (0.03)	0.001 (0.34)	−0.001 (0.81)	0.001 (0.84)			0.001 (0.70)
Age	0.02 (0.005)*	0.01 (0.011)	0.01 (0.004)*	0.02 (0.004)*	0.02 (0.004)*			0.02 (0.004)*
Control Age	−0.04 (0.06)	−0.03 (0.05)	−0.001 (0.16)	−0.001 (0.82)	0.001 (0.38)			0.001 (0.68)
Mediation	15% (ns)	16% (ns)	ns	ns	ns			ns

Direct: Effect of communication-related measure (predictor) on Social ABCs outcome variable. Age: Effect of baseline age on communication-related measure. Age Controlled: Effect of communication-related measure on Social ABCs variable when controlling for age. Mediation: The full mediating effect of the communication-related measure and age on Social ABCs variables. Values are b (*p*-value) for Direct, Age, and Control Age; the value for Mediation is the percentage of variance accounted for by the mediation model (*p*-value; ns: not significant). *Regression is significant at adjusted p ≤ 0.01 (bold font). ^1^MacArthur Communicative Development Inventories – Words and Gestures (Fenson et al., 2007).

#### MSEL expressive language

MSEL-EL-AE did not directly or indirectly predict change scores for any of the Social ABCs outcome variables. The direct effect for caregiver fidelity approached significance (*p* = 0.04), with an inverse association, but did not meet our conservative critical value of *p* ≤ 0.01.

#### VABS-3 communication – receptive (RC)

VABS-RC-AE significantly predicted change scores for Fidelity and Responsivity. A trend toward an inverse association with Social Orienting (*p* = 0.03) failed to meet our critical *p*-value. Significant results are described further below:

##### Fidelity

The direct effect of VABS-RC-AE on Fidelity was significant (*b* = −0.81, *p* < 0.01). Age did not predict VABS-RC-AE (*b* = 0.09, *SE* = 0.09, *t* = 1.04, *p* = 0.31), but when Age was controlled, the effect of VABS-RC-AE dropped below the critical significance level (*b* = −0.72, *SE* = 0.29, *t* = 2.46, *p* = 0.02). The indirect effect of VABS-RC-AE on Fidelity through Age was not significant (Effect = −0.09, 95% CI [−0.30, 0.08]). However, the full mediation model was statistically significant and accounted for 21% of the variance in Fidelity, suggesting that VABS-RC-AE significantly predicted caregivers’ gains in implementation fidelity.

##### Responsivity

The total effect of VABS-RC-AE on Responsivity was significant (*b* = 0.03, *p* < 0.01), even when Age was controlled (*b* = 0.03, *SE* = 0.01, *t* = 2.93, *p* < 0.01). VABS-RC-AE was not predicted by Age (*b* = 0.06, *SE* = 0.09, *t* = 0.64, *p* = 0.52). The full mediation model accounted for 17% of the variance in Responsivity but was not statistically significant. The indirect effect through Age was not significant (Effect = −0.06, 95% CI [−0.01, 0.003]), suggesting that VABS-RC-AE significantly predicted toddlers’ change in Responsivity.

#### VABS-3 communication – expressive (EC)

VABS-EC-AE significantly predicted change scores for Fidelity but no toddler outcomes.

##### Fidelity

The total effect of VABS-EC-AE on Fidelity was significant (*b* = −1.04, *p* = 0.003), remaining significant when Age was controlled (*b* = −0.90, *SE* = 0.339, *t* = 2.73, *p* = 0.001). The full mediation model was statistically significant, accounting for 23% of the variance in Fidelity. The indirect effect of VABS-EC-AE on Fidelity through Age was not significant (Effect = −0.14, 95% CI [−0.38, 0.06]), suggesting that VABS-EC-AE was a significant predictor of caregivers’ change in implementation fidelity.

#### MacArthur CDI – words understood (CDI-WU)

Parent ratings on the CDI-WU significantly predicted change scores for Responsivity. There was a trend toward an association with Fidelity (*p* = 0.02), but this effect did not meet the conservative threshold for significance of *p* < 0.01. Finally, the full model for Child Smiling, with CDI-WU accounting for 13% of the variance, approached, but also did not meet the significance threshold established for the study.

##### Responsivity

The direct effect of CDI-WU on Responsivity was significant (*b* = 0.002, *p* = 0.001), remaining significant when controlling for Age (*b* = 0.002, *SE* = 0.0004, *t* = 3.72, *p* = 0.001). The full mediation model was statistically significant and accounted for 24% of the variance in Responsivity. The indirect effect of CDI-WU on Responsivity through Age was not significant (Effect = −0.0001, 95% CI [−0.0003, 0.0002]), suggesting CDI-WU was a significant predictor of change in Responsivity.

#### MacArthur CDI–words spoken (CDI-WS)

##### Fidelity

The direct effect of CDI-WS on Fidelity was significant (*b* = −0.05, *p* < 0.01). However, Age significantly predicted CDI-WS (*b* = 0.02, *SE* = 0.006, *t* = 3.00, *p* = 0.005) and when Age was controlled, the direct effect of CDI-WS fell below the critical significance level (*b* = −0.04, *SE* = 0.02, *t* = 1.91, *p* = 0.06) and the full mediation model failed to reach significance. The indirect effect of CDI-WS on Fidelity through Age was not significant (Effect = −0.01, 95% CI [−0.03, 0.01]), suggesting that Age mediated the relation between CDI-WS and Fidelity.

##### Responsivity

The total effect of CDI-WS on Responsivity approached, but was not significant (*b* = 0.001, *p* = 0.03). Moreover, although the full mediation model accounted for 16% of the variance in Responsivity, the model failed to reach statistical significance. The indirect effect of CDI-WS on Responsivity through Age was also not significant (Effect = −0.0004, 95% CI [−0.0001, <0.0001]), suggesting CDI-WS was not a significant predictor of Responsivity.

## Discussion

Intervention research has only begun to understand the extent to which a child’s early developmental proficiencies might influence their response to treatment, with particular knowledge gaps in the toddler years, which are often characterized by rapid developmental changes. This paper contributes to the field by identifying communication-related predictors of outcomes in response to a caregiver-mediated intervention for toddlers with emerging autism.

The largest effects emerged for caregivers’ implementation fidelity. Specifically, toddlers’ baseline communicative functioning (i.e., their everyday application of both receptive and expressive abilities) predicted their caregivers’ mastery of program strategies. Consistent with hypotheses, this was an inverse association, meaning that toddlers with lower communication functioning at program entry had caregivers who made larger gains in strategy use while participating. One possibility for this finding is that, when interacting with a toddler who experiences greater communication challenges, some caregivers may have had more difficulty knowing how to engage them prior to learning the Social ABCs, thus leaving room for substantive growth of skills with intervention. Conversely, learning the program strategies may be easier with children who are at earlier stages of communication development. For example, it may be more straightforward to focus on core techniques such as building clear opportunities for toddler responding than to implement the strategies in the context of elaborate activities such as imaginary play, which may be the situation for toddlers with more advanced communicative functioning. Another plausible explanation is that caregivers may be particularly motivated to learn the enhancing techniques when they notice greater developmental delays in their young children. We have yet to demonstrate an association between caregiver motivation (or “buy-in”) and their program uptake, but such an association appears to be consistent with findings reported by Schertz et al. (2020) regarding increased self-efficacy for caregivers when enabled to take a lead role in their child’s learning. These associations warrant careful examination in future studies and may be particularly relevant in the emerging discourse about pre-symptomatic intervention (e.g., see Grzadzinski et al., 2021). We have added a measure of caregiver buy-in to our ongoing work which will allow us to begin exploring this phenomenon.

Among toddler outcomes, increased responsivity to caregivers had the strongest predictors. Significant predictors emerged for caregiver-reported indices of toddlers’ *receptive* (communication and words understood) but not *expressive* abilities. These predictions were in a positive direction, meaning that higher baseline receptive proficiencies predicted steeper gains in responsivity, with toddlers’ comprehension of words accounting for almost one-quarter (24%) of the variance in outcomes. It was contrary to our hypotheses that these associations were restricted to toddlers’ *receptive* abilities, particularly because the intervention, at least nominally, targets toddlers’ *expressive* (vocal) communication-related skills (along with social attention and affect-sharing), and most related work has focused on expressive abilities predicting outcomes. However, receptive abilities may be associated with other ‘learning readiness’ skills such as child attention (and/or joint attention; Bottema-Beutel, 2016), motivation, and participation, any of which might be essential components of a child’s responsiveness to intervention. To our knowledge, this is the first intervention study with autistic toddlers to demonstrate the predictive power of *receptive* (rather than *expressive*) communication-related skills.

Finally, the trend toward a positive association between baseline word comprehension (interacting with age) and toddler smiling, warrants some discussion. The full model accounted for 13% of the variance but failed to meet our stringent threshold for significance. Such a pattern suggests that older toddlers, with larger receptive vocabularies, tended to show greater increases in smiling during the intervention. Our interest in smiling is motivated by findings from longitudinal research showing that, as early as 12 months of age, toddlers who subsequently receive an autism diagnosis express less social smiling than their non-autistic peers (Zwaigenbaum et al., 2005). This is relevant given that positive emotions play a fundamental role in language development (Hohenberger, 2011), social learning, and early relationship-building (Seehagen et al., 2024). Increased smiling during the course of the program may also be an indication that the child is enjoying the interaction, which would support the program’s emphasis on building learning opportunities through activities and interactions that are motivating for the child (Brian et al., 2016). The importance of fostering positive affect is underscored by previous findings that high positive affect, combined with other factors, predicted greater response to intervention for autistic preschoolers (Fossum et al., 2018). In the current study, the full mediation model approached significance, warranting further attention in future work to more fully understand the nature of the associations between toddler communication-related skills and positive affect.

The current findings contribute to an evolving understanding of how early communication-related skills impact response to intervention. Findings revealed significant predictions for caregiver fidelity and toddler responsivity, based on toddlers’ baseline skills, but these occurred in opposite directions. Moreover, findings also failed to show associations for several variables. Notably, expressive communication-related skills, as measured in the current study, did not significantly predict any of the toddler outcomes. As such, findings do not support the use of a toddler’s expressive abilities to guide clinical decisions about their inclusion or exclusion in the program. The current sample included toddlers from a wide range of abilities including those with expressive language and communication skills at a 4-month level and no words spoken or understood. Half the sample (34/67 = 50.1%) began the program with an expressive language age equivalent at or below a 12-month level (nine of whom started the program at or below a 6-month level). Notably, however, the sample also included toddlers with strong communication-related skills at study entry (i.e., expressive language at a 36-month age-level, age-appropriate communication skills, and close to 400 words understood and used). In light of this wide range of abilities across participants, the limited prediction of outcomes other than fidelity and responsivity, together with the lack of association between expressive abilities and any of the child outcomes, suggests that the program is a good fit for toddlers across a wide range of abilities.

It should not be surprising that young children with diverse strengths and needs benefitted from the program in different ways. For example, receptive communication positively predicted responsivity, but trended in an inverse direction for social orienting, suggesting that toddlers with lower communication abilities may have benefitted relatively more from the non-verbal aspects of the program. The ability to orient one’s attention to a social partner is arguably a necessary element of joint attention, which is a well-established marker of early autism and a predictor of later development across various indices of language (Mundy et al., 1990; Kasari et al., 2012; Bottema-Beutel, 2016). Adamson et al. (2019), for example, showed that “coordinated joint engagement,” which entails the toddler explicitly attending to the caregiver during a shared activity, is a stronger predictor of expressive language development than is joint attention. In that study, toddlers who were not yet using spoken words had particularly notable challenges with joint engagement, and the acquisition of first spoken words was associated with gains in joint engagement (i.e., “Moving from not talking to talking seemed to kindle a developmental transformation of joint engagement,” Adamson et al., 2019, p. 16). This appears to be a reciprocal relationship, with joint engagement establishing the opportunity for ongoing language learning. Although the current design did not allow for such analyses, it is possible that toddlers beginning the Social ABCs with lower communication proficiencies needed first to establish social orienting before other learning could be optimized. As a field, intervention research will benefit from acknowledging that a unitary outcome may not be a reasonable expectation for all participants, recognizing the value of personalized treatment pathways and individualized outcomes.

The issue of measurement has been identified as a priority in intervention research (Grzadzinski et al., 2023). In the current study, different measures were used to capture varied aspects of communication-related skills, including a caregiver interview that measured toddlers’ everyday communication, a parent-report questionnaire to capture toddlers’ word use and comprehension, and a direct clinical assessment of expressive language. Unexpectedly, the parent-reported measures had more predictive power than the clinical assessment measure selected for the study. This highlights the importance of obtaining information from multiple sources when identifying children’s learning strengths and needs. However, caution is warranted against the exclusive use of parent-report measures, in light of evidence that caregivers may be inclined to overestimate the receptive language proficiency of their child due to the child’s use of contextual, non-verbal cues that commonly occur in interactions with caregivers (Luyster et al., 2008). Such cautions may be particularly relevant in the context of CMIs, where caregivers cannot be blinded to treatment condition and thus may be particularly vulnerable to an expectancy effect or unconscious bias (e.g., detection bias [Sandbank et al., 2023], or effort justification bias).

### Limitations

#### Clinical assessment measure

Measurement of toddlers’ expressive language development using the MSEL, rather than via a specific language measure (such as the PLS; Zimmerman et al., 2011) may limit findings. Previous work showed that the PLS-4 yielded information that was consistent with the MSEL both before and following participation in the Social ABCs in a protocol that was consistent with the current project (Brian et al., 2017), but future studies may benefit from the use of more specific measures of early language abilities.

Further, this study had no direct clinical assessment of receptive language skills, limiting the ability to draw conclusions about the role of formal measures of receptive language on program outcomes. This decision was made, in part, to be sensitive to young children’s often limited tolerance for lengthy assessments and supported by evidence of greater clinician-parent concordance for expressive than receptive language in autistic toddlers (Federico et al., 2021). This limitation may be mitigated by previous findings of strong associations between expressive and receptive language development in toddlers at elevated likelihood for autism as well as in typically developing controls (Longard et al., 2017). Moreover, in light of evidence of a relative expressive language advantage early in development in autism (Volden et al., 2011), the current findings can be viewed as a conservative evaluation of the influence of overall language proficiency on response to the program. Although the focus on expressive (mostly “vocal/spoken”) language, for the clinical assessment, limits our ability to fully characterize a child’s *overall* language abilities, expressive language may be the most feasible marker for future clinical applications (i.e., relatively easier to elicit from a natural language sample than receptive language). We further acknowledge that the Social ABCs focuses on *vocal* expression, rather than on nonverbal communication, such as gestures, thus limiting findings to this modality. Notably, inclusion of parent-reported indices of receptive communication and understanding of words did allow us to explore at least some aspects of children’s receptive communication-related abilities in the current sample, with these receptive measures emerging as the only predictors of toddler outcomes. Finally, the use of augmentative and alterative communication (AAC) systems has not been explored in the Social ABCs, so current findings cannot be extrapolated to the use of such supports (but see recent work by Frolli et al., 2022 on the use of different AAC strategies with non-speaking autistic toddlers).

#### Focus on communication-related skills

The exclusive focus on communication-related skills as putative predictors means that the role of other possible predictors and interactions with socio-demographic variables was not examined in the current study. These decisions were made given the relatively modest size of the sample, together with the specific objective of examining communication-related predictors that can be used to inform clinical decision-making. More nuanced examination of other child-level and caregiver-level factors, and their interactions with socio-demographic variables is warranted in future work with larger samples. Child-level characteristics, intervention approaches, and parental involvement (Wong et al., 2014) are all factors that likely interact in complex ways with systemic barriers that limit program access, such as socio-demographic disadvantages (e.g., poverty, housing insecurity, lack of educational opportunities; Jonkman et al., 2023), adding to the complexity of this work.

#### Role of sample characteristics

The role of caregiver-level factors (other than fidelity) was not explored, although previous Social ABCs work has been unable to find associations between outcomes and family factors such as marital status, languages spoken, caregivers’ ethnicity, occupation, or education (Brian et al., 2022b). Efforts to enroll a diverse sample resulted in almost 45% of caregivers identifying as racialized (BIPOC) and roughly equal representation across major educational levels (high school, college, undergraduate university). These rates appear to address previous concerns about the lack of diversity in predominantly White and higher-educated samples typically reported in caregiver-mediated intervention studies (e.g., see Trembath et al., 2019, for a systematic review). However, the current sample also had a relatively high proportion of caregivers who were married or in a common-law relationship (~74%), over 13% had a graduate degree, and all participants had to comprehend some English, given that the coaching took place only in English. Trembath et al. (2019) identified practical barriers to accessing caregiver-mediated programs, such as limited time/availability, travel-related limitations, and financial pressures. It is plausible that caregivers in married or common-law relationships may face fewer practical barriers to taking on opportunities for caregiver learning (e.g., childcare, scheduling, travel), and those with relatively higher educational attainment might experience other related advantages such as reduced economic hardship (Campbell, 2015), employment with more flexible work schedules, and relatively strong learning and literacy skills (Lowder et al., 2022). While such associations have yet to be fully articulated, the current findings may not be generalizable to caregivers with educational, marital, or cultural backgrounds that were not represented.

#### Terminology

Finally, we acknowledge that the field continues to struggle with consistent definitions of “minimally verbal” or “nonverbal” – in some cases, these terms refer only to an individual’s use of *spoken* language (where the term “non-speaking” may be preferred; Aidonopoulou-Read, 2025). Recent advocacy efforts are increasingly recommending consideration of an individual’s total communication profile, which may include language comprehension and nonverbal means of expression such as gestures, writing, or use of alternative and augmentative communication (AAC) supports (Aidonopoulou-Read, 2025). In the current study, we have not declared a specific definition of “minimally verbal,” partly due to the young age of participants. Also, instead of treating communication proficiency as a dichotomous variable (“minimally verbal” or otherwise), we have taken the approach of examining communication-related skills on a continuum. The current paper examined two nonverbal behaviors (i.e., social orienting, and smiling) that could be viewed as forms of (or precursors to) communication, but we did not explore the use of AAC devices, since, to our knowledge, none of the toddlers in the current study were using AAC supports at the time of their participation.

## Conclusion

Some, but not all, indices of toddlers’ pre-intervention communication-related skills were informative for predicting response to the intervention. Specifically, lower (expressive and receptive) communication development in toddlers predicted larger gains in caregivers’ strategy use. Conversely, higher baseline (receptive but not expressive) skills predicted greater gains in toddlers’ responsivity. Predictions occurred in both positive and inverse directions, highlighting the complexity of the relationships between baseline proficiency and outcomes. Starting the program with relative developmental advantages appeared to predict greater gains in toddlers’ vocal responsivity (with a parallel trend for social smiling), whereas starting with relatively lower proficiency appeared to be an advantage for caregivers’ learning, and may also have subtly impacted toddlers’ social orienting. The clinical measure selected for this study did not have any explanatory power, but other clinical measures may be useful for capturing different information.

The current findings may be helpful in informing clinical triage decisions and personalizing intervention approaches. Our results support the use of the Social ABCs with a wide range of toddlers with autism or early signs thereof, with no consistent evidence that the program systematically favors toddlers at either end of a wide range of ability. Clinicians should feel confident in referring toddlers with autism, or related social communication and behavioral differences, to caregiver-mediated early interventions that support social communication development, regardless of the child’s communication-related abilities (whether high or low) at program entry.

Future research will benefit from further exploring the complex interactions among child-, caregiver-, program-, and system-level factors that predict treatment outcomes, with the goal of developing individualized treatment pathways that are informed by evidence. The toddler period is a key stage in development, during which strengthening foundational social communication skills can enhance long-term outcomes (e.g., Pickles et al., 2016; Warlaumont et al., 2014). This highlights the importance of finding the best “fit” between each toddler’s developmental needs, their family and community characteristics, and the type, timing, and intensity of intervention and supports that they receive.

## Data Availability

The datasets presented in this article are not readily available per Research Ethics Board approval. Requests to access the datasets should be directed to jbrian@hollandbloorview.ca.
